# Exploring perspectives, preferences and needs of a telemonitoring program for women at high risk for preeclampsia in a tertiary health facility of Karachi: a qualitative study protocol

**DOI:** 10.1186/s12978-020-00979-8

**Published:** 2020-09-15

**Authors:** Anam Feroz, Sarah Saleem, Emily Seto

**Affiliations:** 1 Department of Community Health Sciences, The Aga Khan University, Stadium Road, PO Box 3500, Karachi, 74800 Pakistan; 2 Institute of Health Policy, Management and Evaluation, Dalla Lana School of Public Health, University of Toronto, Toronto, ON Canada; 3 Centre for Global eHealth Innovation, Techna Institute, University Health Network, Toronto, ON Canada

**Keywords:** Pregnant women, High risk for preeclampsia, Telemonitoring, Home blood pressure monitoring, Qualitative study

## Abstract

**Background:**

In Pakistan, deaths from preeclampsia/eclampsia (PE/E) represent one-third of maternal deaths reported at tertiary care hospitals. To reduce the morbidity and mortality associated with PE/E, an accessible strategy is to support pregnant women at high risk for preeclampsia (HRPE) by closely monitoring their blood pressures at home (i.e., telemonitoring) for the earliest signs of preeclampsia. This could lead to the earliest possible detection of high blood pressure, resulting in early intervention such as through medications, hospitalization, or delivery of the baby. The study aims to explore the perspectives, preferences and needs of telemonitoring (TM) for pregnant women at HRPE in Karachi, to inform future implementation strategies.

**Methods:**

The study will employ an exploratory qualitative research design. The study will be conducted at the Jinnah Postgraduate Medical Centre (JPMC) hospital and Aga Khan University Hospital (AKUH) in Karachi, Sindh, Pakistan. Data will be collected through key-informant interviews (KIIs) and in-depth patient interviews (IDPIs). IDPIs will be conducted with the pregnant women at HRPE who are visiting the out-patient department/ antenatal clinics of JPMC hospital for antenatal check-ups and immunizations. KIIs will be conducted with the obstetricians, Maternal, neonatal and child health (MNCH) specialists and health care providers at JPMC, as well as TM experts from Karachi. Study data will be analyzed through conventional content analysis. Interviews are anticipated to begin in April 2020 and to be completed during the summer of 2020.

**Discussion:**

This is the first study to explore the use of TM program for pregnant women at HRPE in a tertiary health facility in Karachi. The research will help explore perceived benefits associated with the use of a TM program alongside potential facilitators and barriers that may help inform the future implementation of a TM program for pregnant women at HRPE in Karachi.

## Plain English summary

Preeclampsia is a disorder of pregnancy that is characterized by high blood pressure and protein in the urine after 20 weeks of pregnancy. Maternal and neonatal deaths from preeclampsia results from a lack of early identification of women at risk for preeclampsia, transportation difficulties to treatment centers, sub-optimal patient education on this topic, and inability to detect signs of imminent danger to the pregnant individual and baby. Telemonitoring is a prevention strategy where pregnant individuals take blood pressure measurements at home through home blood pressure machines and these readings are sent to their healthcare providers. It can enable the earliest detection of high blood pressure, resulting in early intervention such as, medications, management hospitalization, or delivery of the baby. The TM platform includes a smartphone app that automatically transfers blood pressure readings wirelessly from a blood pressure home monitor and sends automated alerts to providers and self-care instructions to the patient based on their readings. The study aims to explore perspectives of health care experts and pregnant women on the use of telemonitoring for pregnant women at HRPE in Karachi. The findings will provide a better understanding of study participants’ attitudes and readiness towards the use of a TM program that is intended to be implemented at JPMC.

## Background

Preeclampsia is a disorder of pregnancy that is characterized by high blood pressure and protein in the urine after 20 weeks of pregnancy. It is associated with high maternal mortality, preterm birth, pregnancy loss, and stillbirth [1]. Women with preeclampsia are at increased risk for stroke and damage to vital organs such as kidneys, liver, and brain. In severe cases, preeclampsia can lead to eclampsia which is the presence of seizures [1]. This condition could also lead to the separation of the placenta from the uterus (referred to as placental abruption) [1]. Infants born preterm due to preeclampsia are at higher risk of some long-term health issues including learning disorders, cerebral palsy, epilepsy, deafness, and blindness [1, 2]. Hypertensive disorders of pregnancy contribute to 14% of all maternal deaths, the majority of which occur in low- and middle-income countries [3].

Pakistan has one of the highest maternal mortality ratios worldwide at 178/100,000 live births [4, 5] and neonatal mortality ratios at 42/1000 live births [6]. Eclampsia accounts for more than 12% of direct maternal deaths [7]. Deaths from preeclampsia/eclampsia represent one-third of maternal deaths reported at tertiary care hospitals [8]. Maternal and neonatal morbidity and mortality from preeclampsia results from a lack of early identification of women at risk for preeclampsia, transportation difficulties to treatment centers, sub-optimal patient education on this topic, and inability to detect signs of imminent danger to the pregnant individual and baby [8].

To meet the targets of the United Nations’ Sustainable Development Goal 3 by 2030 (maternal mortality ratio < 70/100,000 live births; neonatal mortality ratio < =12/1000 live births) [9], strategies are required to decrease the rate of mortality from preeclampsia/eclampsia. An accessible strategy to meet this goal is to support pregnant women at HRPE by closely monitoring their blood pressure at home (i.e. TM) in order to detect the earliest signs of preeclampsia. TM is a prevention strategy where pregnant individuals take blood pressure measurements at home through home blood pressure machines and these readings are sent to their healthcare providers. Automated clinical alerts can also be generated based on the patient’s readings, as well as self-care messages for the patient. TM can enable the earliest detection of high blood pressure, resulting in early intervention such as, medications, management hospitalization, or delivery of the baby [10, 11].

Several retrospective and prospective studies have recommended the use of home blood pressure monitoring (HBPM) in pregnancy because of its potential benefits including fewer hospital visits, better blood pressure control, and cost savings [12–15]. A scoping review conducted in 2019 found 16 unique TM interventions from 20 studies [12, 14, 16–33] for patients at high-risk for hypertensive disorders of pregnancy, but these studies have not translated to sustained programs [34]. The scoping review concluded that TM for hypertension during pregnancy was feasible, convenient, and cost-effective. Most TM interventions have been implemented in high-income countries including UK, USA, and Belgium. There is little evidence available on the use of a TM platform for pregnant women in LMICs. Thus, the feasibility and effectiveness of TM interventions to support pregnant women at HRPE in a low-middle-income setting may be different due to differing patient demographics, healthcare services available, cultural considerations, budgetary constraints and clinical workflows. Implementing a TM program for pregnant women at HRPE in Karachi, Pakistan would require a thorough understanding of perceived benefits associated with the use of a TM program alongside potential facilitators and barriers that may help inform the future implementation of such a TM program in a tertiary health facility of Karachi. In addition, exploring the readiness of the deployment site towards the use of a TM program for pregnant women at HRPE, is particularly essential to ensure engagement of the deployment site during the implementation phase. Prior to implementing a similar TM program in Karachi, Pakistan, it is crucially important to explore the perspectives and readiness of the healthcare professionals’ and end users (pregnant women at HRPE) regarding the use of TM for preeclampsia.

### Study aim & objectives

The aim of this study is to explore the perspectives, preferences and needs of TM for pregnant women at HRPE in Karachi, to inform a potential future implementation. In particular, a smartphone-based TM platform developed in Canada is being considered as a possible system for TM of pregnant women at HRPE in Karachi [35–44]. The TM platform includes a smartphone app that automatically transfers blood pressure readings wirelessly from a blood pressure home monitor and sends automated alerts to providers and self-care instructions to the patient based on their readings. The specific objectives of this study are:

1. To explore pregnant women’s perspective, preferences and needs of TM, and in particular smartphone-based TM, for pregnant women at HRPE in Karachi.
2. To explore the perspectives of obstetricians, healthcare providers (doctors, nurses and midwives), Maternal, neonatal and child health (MNCH) specialists, and TM experts on the use of TM for pregnant women at HRPE in Karachi.

## Methods

### Study design

This formative research will employ an exploratory qualitative research design using semi-structured interviews and a purposive sampling approach. The data collection methods for this formative research will involve key-informant interviews (KIIs) and in-depth patient interviews (IDPIs).

### Study setting

The study will be conducted at Jinnah Postgraduate Medical Centre (JPMC) hospital and Aga Khan University Hospital (AKUH) Karachi, Sindh, Pakistan. JPMC serves over one million patients of low-socio-economic status per year from Karachi, Interior Sindh, Baluchistan and other areas. Each year, approximately 60,000 patients are admitted, with 30,000 surgeries and 15,000 deliveries, all free of charge. JPMC is a 1650-bed tertiary care public sector hospital with over 31 departments [45]. Pregnant women at HRPE will be identified from the out-patient department (OPD) area where women wait for several hours to be seen by their healthcare providers.

AKUH was established in 1985 as one of the principal teaching sites for nurses and doctors from the university’s School of Nursing and Midwifery and the School of Medicine. AKUH’s multidisciplinary approach to diagnosis and care ensures a continuum of safe and high-quality care for patients, with all services under one roof. Key informants’ such as obstetricians, gynecologists, and few TM experts will be identified from relevant departments of Aga Khan University.

### Data collection methods

#### Key-informant interviews (KIIs)

We will invite ‘key informants’ such as obstetricians, healthcare providers, MNCH specialists, and TM experts to understand their perspectives, preferences and needs regarding use of TM for pregnant women at HRPE.

The KIs will be identified from AKUH, JPMC, Non-governmental organizations (NGOs) and the Aga Khan Development Network Digital Health Resource Center (AKDNdHRC) and other relevant institutions. A few KIIs with obstetricians and healthcare providers (doctors, nurses and midwives at JPMC) will be arranged at JPMC. A few obstetricians and MNCH specialists will be identified from AKUH and NGOs. Experts in the field of TM will be identified and interviewed from AKUH and the AKDNdHRC to understand the technological feasibility of TM intervention in Karachi, Pakistan. KIs will be invited to participate in the qualitative study via email. KIs will be requested to sign consent forms (Additional file 1) before the interview begins, in which they will agree that the interview can be audio-recorded and written notes can be taken by a note-taker to record any relevant observations. It is anticipated that the interviews will be conducted in the languages of Urdu or Sindhi. Initially, the KII will involve discussion around causes of PE and routine obstetric care for PE. Later, the discussion will move towards exploring participant views towards use of TM for pregnant women at HRPE. Finally, the interview will explore perceived facilitators and barriers for implementation of TM, including smartphone-based TM. We anticipate that 13–15 participants will be recruited for KIIs, but additional interviews will be conducted until data saturation. Each KII will take approximately between 30 and 45 min.

#### In-depth patient interviews (IDPIs)

IDPIs will be conducted with pregnant women at HRPE who are visiting the OPD/antenatal clinics of JPMC hospital for antenatal check-ups and immunizations. Pregnant individuals at HRPE will be identified using National Institute for Health and Care Excellence (NICE) guidelines. Considering the cultural and ethical sensitivity of preeclampsia, the research will not have interviews with high risk pregnant women who are admitted at the emergency department and those who are in-patients. Pregnant women at HRPE will be identified from the OPD area where women wait for several hours to be seen by their healthcare providers. Recruiting pregnant women at HRPE, from a busy OPD area would be challenging and therefore healthcare providers at JPMC involved in antenatal clinic such as midwives, nurses and technicians, will be requested to support identification of pregnant individuals at HRPE. The study coordinator will then explain the study objectives and procedures to eligible pregnant individuals at HRPE and will obtain written consent for participation in the study (Additional file 1). If the participant is unable to read the consent form, then the study coordinator will explain the consent form verbally in their local language. Participants who are unable to write their names will be asked to provide a thumbprint to mark their consent to participate. Women who provide consent will be asked to move to a separate private room near the OPD area of JPMC, to conduct IDPIs with high-risk pregnant women without any disruption. Caregivers would be able to accompany the patient during the interviews if the patient prefers. The interview will be conducted by a trained researcher (study Principal Investigator) who is experienced in conducting qualitative research. Participants will be assured that their anonymity will be maintained, and no identifying information will be included on the transcripts. The interview will involve a general discussion about healthy pregnancy related to being HRPE, knowledge about preeclampsia (PE), causes of PE, perceptions towards use of TM for pregnant women at HRPE, perceived benefits of TM, potential limitations or concerns related to TM for PE, and feasibility of smartphone-based TM. We anticipate that 10–15 IDPIs will be conducted with pregnant women at HRPE (Table 1). However, additional IDPIs will be conducted until data saturation. Each IDI will approximately take between 30 and 45 min.

**Table 1:** Study Participants for KIIs and IDPIs

**Participants for Key-informant Interviews (KIIs)**	**Sample Range**
Obstetricians at JPMC & AKUH	03–05 KIIs
Healthcare providers at JPMC (Doctors/ nurses/ midwives)	03–05 KIIs
Maternal, neonatal and child health specialists at NGOs and AKUH	03–05 KIIs
Telemonitoring experts at AKDNdHRC	03–05 KIIs
**Participants for In-depth Patient Interviews (IDPIs)**	**Sample Range**
Pregnant women at high risk of pre-eclampsia (HRPE) who are visiting the OPD/antenatal clinics of JPMC hospital for antenatal check-ups and immunizations	10–15 IDIs

### Study participants

Purposive sampling technique will be used to identify and recruit KIs and IDPIs participants for the study. Detailed eligibility criteria are given below:

#### Eligibility criteria

##### Inclusion criteria

Pregnant Individuals at HRPE (IDPIs)

- Based on the National Institute for Health and Care Excellence (NICE) guidelines, pregnant individuals at HRPE will be defined as those who have one high risk factor or more than one moderate risk factor for preeclampsia [46].

High risk factors include:

- Hypertensive disease in a previous pregnancy
- Chronic kidney disease
- Autoimmune disease, such as systemic lupus erythematosus or antiphospholipid syndrome
- Type 1 or type 2 diabetes
- Chronic hypertension

Moderate risk factors include:

- First pregnancy
- Age 40 years or older
- Pregnancy interval of more than 10 years
- Body mass index (bmi) of 35 kg/m2 or more at first visit
- Family history of preeclampsia
- Multi-fetal pregnancy
- Pregnant individuals at HRPE, who are willing to give consent to participate in the study and are able to speak either Urdu or Sindhi language

##### Key-informants

- Healthcare professionals who are directly or indirectly involved in the care of pregnant individuals at HRPE such as, obstetricians, healthcare providers (doctors, nurses and midwives at JPMC), MNCH specialists, and TM experts

##### Exclusion criteria

- The study will not include women who are newly diagnosed as HRPE (i.e., recruiting during follow-up visits at least 1 week after being diagnosed as HRPE), as they may not have had time to experience or reflect on the associated challenges.
- Considering the cultural and ethical sensitivity, the research will not include interviews with high risk pregnant women who are admitted to the emergency department and in-patient areas for treatment purpose.

### Data collection procedure

Separate semi-structured interview guides have been developed for KIIs and IDPIs (Additional files 2 & 3). Both the interview guides will be pilot tested with a non-study sample (2 KIIs & 2 IDPIs) who have the same characteristics as the study sample. The pilot testing will offer evidenced-base guidance to improve data collection guides [47].

To mitigate bias, the researcher will first explore patient participants’ views towards the use of TM for pregnant women at HRPE, perceived benefits of TM, potential limitations or concerns related to TM for PE, and implementing TM for PE into clinical practice. Then, the researcher will show a power-point presentation to the patient participants to introduce the idea of TM for pregnant women who are at HRPE. The presentation will be made in the participant’s local language (Urdu or Sindhi) and will mainly include graphics and illustrations to help ensure participants understand the concepts of TM and HBPM. The presentation will provide an overview on the disease scenario of preeclampsia, information on routine obstetric care of PE, and description on the potential future use of TM for high risk pregnant women. Later, the researcher will explore participants’ perspectives, preferences and needs regarding use of a TM program for women at HRPE in Karachi. It is possible that the pregnant women at HRPE may feel anxiety from being asked questions about their experiences and knowledge of managing their HRPE condition. To address that, the investigator will explain to the participants the study objectives and answer any queries arising from the interview. At the end of the interview, a PowerPoint presentation will be given to patient participants to provide them with an understanding of preeclampsia, its consequences, and simple ways to manage the condition through regular follow-ups and blood pressure monitoring. Interviews are anticipated to begin in April 2020 and to be completed during the summer of 2020.

### Data analysis

The audio recordings from the interviews will be transcribed and will then be translated into English for conventional content analysis [48–50]. No identifying characteristic will be included in the transcriptions. The transcripts will be uploaded into NVivo 12 Plus software to enable easy and organized retrieval of data for analysis. Transcripts will be read several times to develop an interpretation of the participants’ perception regarding the use of TM for pregnant women at HRPE. This will involve an iterative process where data will be coded, compared, contrasted and refined to generate emergent themes. This iterative process will involve revisiting the data or going back and forth repeatedly on the data to address the data gaps and to seek information to saturate the gaps in subsequent contacts with new research participants [51]. The transcribed text will be divided into ‘meaning units’ which will be later shortened and labeled with a ‘code’ without losing the study context. Codes will be then analyzed and grouped into similar categories. In the final step, similar categories will be assembled under sub-themes and main themes. Two independent investigators will perform the coding, and category creation, and discrepancies will be resolved to through discussion until a consensus is reached. To gain a more complete understanding of the perspectives, preferences and needs of TM for women at HRPE, the themes from the KIIs and IDPIs will be compared and contrasted.

### Ethical considerations

Ethical approval for this study has been obtained from the Aga Khan University Ethical Review Committee (AKU-ERC) – [2020–2153-8519].

## Discussion

This is the first study to explore the use of TM program for pregnant women at HRPE in a tertiary health facility in Karachi. This research will help explore perceived benefits associated with the use of a TM program, as well as potential facilitators and barriers that may inform the future implementation of a TM program for pregnant women at HRPE in a tertiary health facility in Karachi. Specifically, the findings of the qualitative study will provide a better understanding of study participants’ attitudes and readiness towards the use of a TM program that is intended to be implemented at JPMC. This study would inform an implementation blueprint, by suggesting tailored strategies for future successful implementation of TM programs across Pakistan.

## Supplementary information

**Additional file 1.** Informed Consent.

**Additional file 2.** Semi-Structured Interview Guide for KIIs.

**Additional file 3.** Semi-Structured Interview Guide for IDPIs.

## Data Availability

Materials described in this paper pertain to the study protocol only and there are no raw data reported. The datasets will be collected and analyzed and can be made available from the corresponding author on reasonable request. Interview guides developed for this study protocol are included as Additional files 2 & 3.

## Abbreviations

HBPM: Home blood pressure monitoring
HRPE: High risk for preeclampsia
IDPIs: In-depth interviews
KIIs: Key-informant interviews
MNCH: Maternal, neonatal and child health
TM: Telemonitoring
AKDNdHRC: Aga Khan Development Network Digital Health Resource Center
NGOs: Non-governmental organizations
AKUH: Aga Khan University Hospital Karachi
JPMC: Jinnah Postgraduate Medical Centre
