# Supplementary material for: Exploring perspectives, preferences and needs of a telemonitoring program for women at high risk for preeclampsia in a tertiary health facility of Karachi: a qualitative study protocol
Source: Reprod Health. 2020 Sep 15;17:135. doi: 10.1186/s12978-020-00979-8 (PMC7491177; doi:10.1186/s12978-020-00979-8)
Supplement: Supplementary file 1 — Additional file 1. Informed Consent. [file 12978_2020_979_MOESM1_ESM.docx]

**Appendix I: Informed Consent**

| **Project Information** | |
| --- | --- |
| Principal Investigator: Ms Anam Feroz | Organization: Department of Community Health Sciences, Aga Khan University |
| Other Investigators: Dr Sarah Saleem | Organization: Department of Community Health Sciences, Aga Khan University |
| Other Investigators: Dr Emily Seto | Institute of Health Policy, Management and Evaluation, Dalla Lana School of Public Health, University of Toronto, Toronto, ON, Canada  Centre for Global eHealth Innovation, Techna Institute, University Health Network, Toronto, ON, Canada |

Date (dd/mm/yyyy): __ __ /__ __/__ __ __ __

**INTRODUCTION**

The research is titled ‘Exploring perspectives, preferences and needs regarding use of telemonitoring program for high risk preeclampsia (HRPE) women in Pakistan: a qualitative study’. This qualitative exploratory research aims to explore perceptions of study participants regarding use of telemonitoring for pregnant individuals at HRPE in Pakistan, to support future implementation. Prior implementation of telemonitoring program, predominantly in Pakistan, we would like to explore and understand perspective, preferences and needs regarding use of telemonitoring for pregnant individuals at HRPE among clinicians, public health experts with MNCH specialty, health technology experts and high-risk pregnant women. The results of the interview will help us better understand the perceived benefits associated with the use of telemonitoring program alongside potential facilitators and barriers that may leverage or influence the future implementation of telemonitoring program, for pregnant individuals at HRPE, in clinical practice. Your participation is voluntary (your choice).

**PROCEDURES**

The study will employ an exploratory qualitative research design. The study will be conducted at the Jinnah Postgraduate Medical Centre (JPMC) hospital in Karachi, Sindh, Pakistan. The data collection method will consist of key-informant interviews (KIIs) and in-depth interviews (IDIs).

IDIs will be conducted with the pregnant women at HRPE who are visiting the OPD/antenatal clinics of JPMC hospital for antenatal check-ups and immunizations. IDIs will take around 30 to 40 mins. To avoid any bias, the researcher will first explore participants’ views towards the use of TM for pregnant women at HRPE, perceived benefits of TM, potential limitations or concerns related to TM use for PE, and implementing TM for PE into clinical practice. Then, the researcher will show a power-point presentation to the study participants to introduce the idea of TM for pregnant women who are at HRPE. Later, the researcher will explore participants’ perspectives, preferences and needs regarding use of a TM program for women at HRPE in Karachi.

KIIs will be conducted with the clinicians, public health experts with MNCH specialty and health technology experts from Karachi. KIIs will take around 30 to 40 mins.

Interviews will be recorded for the transcription purpose. The aim of the KIIs and IDIs is to explore broadly study participants’ attitudes and readiness towards the use of telemonitoring program, for pregnant women at HRPE.

**POSSIBLE RISKS OR DISCOMFORT**

Your participation will not pose any risk to you.

**POSSIBLE BENEFITS**

There is no immediate benefit to you for participating in this study. The information shared by you will help us to understand the facilitatory and inhibitory factors perceived by study participants for the future implementation of telemonitoring program for pregnant individuals at HRPE. This information will eventually help us to recognize the role of telemonitoring for preeclampsia, a global public health issue, from a perspective of pregnant women at HRPE, health care providers, clinicians, and health technology experts.

**FINANCIAL CONSIDERATIONS**

There is no financial compensation for your participation in this research. No monetary inducements will be given to study participants for participation. Only refreshments for study participants: Refreshments will be provided to study participants, particularly pregnant women, who will participate in IDIs.

**AVAILABLE TREATMENT ALTERNATIVES**

This study is not going to experiment or test any treatment or drugs.

**CONFIDENTIALITY**

Your identity in this study will be treated as confidential. The results of the study, including views expressed by you, may be published for scientific purposes but will not give your name or include any identifiable references to you. However, any records or data obtained as a result of your participation in this study may be inspected by the sponsor or by AKU ERC members.

**TERMINATION OF RESEARCH STUDY**

You are free to choose whether or not to participate in this study. There will be no penalty or loss of benefits to which you are otherwise entitled if you choose not to participate.

**AVAILABLE SOURCES OF INFORMATION**

Any further questions you have about this study will be answered by the Principal Investigator:

Name: Ms Anam Feroz Phone Number: 021-3484917

**AUTHORIZATION**

I have read and understand this consent form, and I volunteer to participate in this research study. I understand that I will receive a copy of this form. I voluntarily choose to participate, but I understand that my consent does not take away any legal rights in the case of negligence or other legal fault of anyone who is involved in this study.

Name of participant (Printed or typed):

Date:

Signature of participant:

Date:


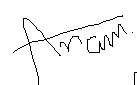
Signature of Principal Investigator: Anam Feroz

Date: March 19, 2020

Signature of person obtaining consent: Salima Ratnani
Date: March 19, 2020
