# Supplementary material for: Exploring perspectives, preferences and needs of a telemonitoring program for women at high risk for preeclampsia in a tertiary health facility of Karachi: a qualitative study protocol
Source: Reprod Health. 2020 Sep 15;17:135. doi: 10.1186/s12978-020-00979-8 (PMC7491177; doi:10.1186/s12978-020-00979-8)
Supplement: Supplementary file 2 — Additional file 2. Semi-Structured Interview Guide for KIIs. [file 12978_2020_979_MOESM2_ESM.docx]

**Semi-Structured Interview Guide for Key-informants**

Key informants: Obstetricians at AKUH & JPMC, healthcare providers including doctors, nurses and midwives at JPMC, maternal, neonatal and child health (MNCH) specialists at AKUH & NGOs, and telemonitoring experts at AKDNdHRC

| S.no | Name (Confidential) | Age | Sex | Designation | Institution | Years of experiences | Specialty |
| --- | --- | --- | --- | --- | --- | --- | --- |
|  |  |  |  |  |  |  |  |
|  |  |  |  |  |  |  |  |
|  |  |  |  |  |  |  |  |
|  |  |  |  |  |  |  |  |
|  |  |  |  |  |  |  |  |
|  |  |  |  |  |  |  |  |
|  |  |  |  |  |  |  |  |

1. **Managing patients at risk of preeclampsia**
   1. What is your current role in managing patients at-risk for pre-eclampsia?

*Probes:*

- How many patients at-risk for pre-eclampsia do you see in your clinic in a month? Has this changed within the last 5 years?
- What are the demographics of these patients?
  1. Can you tell me how patients at-risk for pre-eclampsia are managed at the clinic?

*Probes:*

- What clinical markers are monitored during pregnancy or in the postpartum period?
- Is there a tool (paper-based, digital or other) to assist healthcare staff monitor these patients?
- How do you use this information for their clinical care?
- How do you collect this information and how is it stored?
  1. Do you think it is important for pregnant individuals at HRPE to monitor blood pressure at home?

Probes:

- If yes, why? If no, why?
- How often do you think they should monitor their BP at home?
  1. What education do you provide patients at-risk for pre-eclampsia during clinic visits?

*Probes:*

- Do you recommend self-care behaviors like taking blood pressure self-measurements at home to these patients?
- Do you provide patients with educational material (brochures, pamphlets) or other resources (website, support groups)?
  1. In your opinion, what are there barriers to supporting the self-care activities of patients who are at-risk for pre-eclampsia? What are there barriers to the clinical management of these patients?
  2. What you do think could be done address these barriers?

1. **Perceptions towards use of telemonitoring for pregnant individuals at HRPE**
   1. Do you ever recommend use of telemonitoring to support the self-care activities of pregnant women at-risk for pre-eclampsia?

*Probes:*

- If yes why? How does the telemonitoring work? If no why?
  1. Do you think pregnant women at HRPE would be able to use a telemonitoring program?

*Probes:*

- If yes why? If no why?

2.3 Do you think it would it be acceptable in the community to use a telemonitoring program for pregnant individuals at HRPE?

*Probes:*

- If yes why? If no why?
- How would the family members of pregnant women feel about this being done?
- How would your community feel about this being done?
- How would this be named in your community?
- What kinds of negative misconceptions might start in the community? Do you have any suggestions about ways we could work in your community to address those misconceptions?
  1. In your opinion, how could a telemonitoring system work?

*Probes:*

- What should be part of the telemonitoring innovation?
- More specifically, what features should be part of the innovation?

1. **Perceived (foreseen) benefits and barriers related to telemonitoring use for pregnant individuals at HRPE**
   1. What do you think would be the benefits of telemonitoring for pregnant individuals at HRPE?

*Probes:*

- Women will be able to take blood pressure measurements at home
- Healthcare providers able to track blood pressure readings of women between antenatal visits.
- Earliest possible detection of high blood pressure
- Early treatment
- Preventing complications and deaths associated with PE/E
  1. What are the barriers/concerns for use of a telemonitoring program for pregnant individuals at HRPE?

*Probes:*

- Traditional/cultural beliefs
- Not supported by husband and mother in law
- Costs associated with the telemonitoring program
- Mistrust on the health professionals
- Mistrust on the telemonitoring program
  1. What do you think would be the best method of educating the community, especially high-risk pregnant women, about telemonitoring program?

*Probes:*

- Community discussions
- Counseling of pregnant women during antenatal visits

3.4 What would be needed in order to create a sustained program of TM for pregnant individuals with HRPE?

*Probes:*

- Level of current knowledge (telemonitoring program) among health care workers.
- Acceptability of the new innovation among health care workers.
- Availability of experts to implement telemonitoring program
- Equipment
- Healthcare facilities – good reputation of the facilities implementing telemonitoring program
- Health professionals attitude and preparedness
- Integration of new innovation within the existing health system
- What would need to be put in place in regard to facilities? Equipment? Personnel?
- Is the health system prepared?
- Unprepared health system (lack of equipment, financial limitations)

3.5 Do you think there will be implementation issues? If yes, what would they be?

*Probes:*

- Lack of skills and training
- Logistics
- Cost implications
- Acceptance and governance
- Reluctance from healthcare professionals (increased workload)
