# Supplementary material for: Exploring perspectives, preferences and needs of a telemonitoring program for women at high risk for preeclampsia in a tertiary health facility of Karachi: a qualitative study protocol
Source: Reprod Health. 2020 Sep 15;17:135. doi: 10.1186/s12978-020-00979-8 (PMC7491177; doi:10.1186/s12978-020-00979-8)
Supplement: Supplementary file 3 — Additional file 3. Semi-Structured Interview Guide for IDPIs. [file 12978_2020_979_MOESM3_ESM.docx]

**Semi-Structured Interview Guide for IDIs**

**In-depth interviewees:** IDIs will be conducted with pregnant women at HRPE who are visiting the OPD/antenatal clinics of JPMC hospital for antenatal check-ups and immunizations. Pregnant individuals at HRPE will be identified using National Institute for Health and Care Excellence (NICE) guidelines.

Note: We will not interview women who were just diagnosed with being HRPE as they may not have had time to figure out the challenges and what they need to do for self-care.

| S.no | Name (optional) | Age | Educational level | Profession | Gravida/para | Hx of Preeclampsia/ Hx of being at high risk for preeclampsia  [Yes, No] | Causes of being at high risk for preeclampsia | Pregnancy trimester & week |
| --- | --- | --- | --- | --- | --- | --- | --- | --- |
|  |  |  |  |  |  |  |  |  |
|  |  |  |  |  |  |  |  |  |
|  |  |  |  |  |  |  |  |  |
|  |  |  |  |  |  |  |  |  |
|  |  |  |  |  |  |  |  |  |
|  |  |  |  |  |  |  |  |  |
|  |  |  |  |  |  |  |  |  |

1. **Current knowledge and perceptions about preeclampsia/eclampsia**
   1. What do you know about the disease conditions like preeclampsia/eclampsia?

*Probes:*

- Seizures
- High blood pressure
- Protein in urine
  1. In your opinion what are the causes of preeclampsia/eclampsia?

*Probes:*

- Common beliefs and perceptions about causes of preeclampsia/eclampsia?

1.3 In your previous pregnancy, were you at high-risk for preeclampsia?

*Probes:*

- If yes, what were the reasons?

1. **Current actions to manage preeclampsia condition**

2.1 How are you currently managing your risk for preeclampsia?

*Probes:*

- Specific diet or exercise program
- Seeing a doctor/regular checkup
- Monitoring blood pressure routinely

2.2 Do you have someone (outside JPMC and other clinic staff) who helps you manage your health related to pregnancy?

*Probes:*

Friend, coworker, partner

How they have helped you?

- 1. Have you faced any challenges managing your health related to your pregnancy?

*Probes:*

- If yes, what kind of challenges
  1. In your opinion, what do you think might be helpful in managing the pregnancy care related challenges?

1. **Current access and use of mobile phones and mobile apps**

3.1 Do you own or have access to the following?

- Basic mobile phone
- Smartphone
- Computer
- Tablet
- Internet connection at home

3.2 Are you comfortable using these?

- Basic mobile phone
- Smartphone
- Computer (such as using MS Office)
- Tablet
- Internet (such as emailing and web browsing)

3.3 Have you ever used a mobile health app?

*Probes*

- If yes, which ones? If not, why not?
  1. Do you think that mobile health apps can help you manage your health related to your pregnancy? Why or why not?

*Probes*

- What would this app be able to do?
- Can you picture yourself using this app?

1. **Closely monitoring blood pressures (i.e. telemonitoring) for pregnant women at HRPE**
   1. Do you think it is important to monitor blood pressure at home (in between clinic visits)?

*Probes:*

- If yes, why? If no, why?
  1. What are the best methods to share your blood pressure readings with your healthcare provider?

*Probes:*

- Sharing through WhatsApp
- Bluetooth (automatic transfer)
- Manual entry

Explanation of telemonitoring system will be given to pregnant individuals at HRPE after question 3 through a PowerPoint presentation.

- 1. Would it be useful to have a mobile based-telemonitoring program where your healthcare providers can monitor your condition at home?

*Probes:*

- If yes, why? If no, why?
  1. In your opinion, what features would be helpful to include in a mobile based-telemonitoring system beyond sending blood pressure measurements to the healthcare providers?
  2. Would you be ready to use a telemonitoring program?

*Probes:*

- If yes, why? If no, why?

4.7 Would it be acceptable in the community to use telemonitoring program for pregnant women at HRPE?

*Probes:*

- If yes, why? If no, why?
- How would the family members of pregnant women feel about this being done?
- How would your community feel about this being done?
- How would this be named in your community?
- Do you think rumors might start in the community? If so, what kinds of rumors might start in the community? Do you have any suggestions about ways we could work in your community to address those rumors if they started?

1. **Perceived (foreseen) benefits and barriers related to telemonitoring use for pregnant women at HRPE**
   1. What do you think are the benefits of using telemonitoring for pregnant women at HRPE?

*Probes:*

- Women will be able to take blood pressure measurements at home
- Healthcare providers able to track blood pressure readings of women between antenatal visits.
- Earliest possible detection of high blood pressure
- Early treatment
- Preventing complications and deaths associated with PE/E
  1. What do you think are the barriers/concerns for the use of telemonitoring program for pregnant women at HRPE?

*Probes:*

- Traditional/cultural beliefs
- Not supported by husband and mother in law
- Costs associated with the telemonitoring program
- Mistrust on the health professionals
- Mistrust on the telemonitoring program
  1. Is there anything else you would like to add that we did not talk about?

A PowerPoint presentation will be given to pregnant individuals at HRPE to provide them understanding of preeclampsia condition, its consequences, and simple ways to manage the condition through regular follow-ups and blood pressure monitoring.

Thank you for your time and participation in this study!
